# *aip* Mutant Zebrafish Display Morphological and Transcriptional Changes Associated with Stress-Induced Hematopoiesis

**DOI:** 10.3390/toxics14090807

**Published:** 2026-09-11

**Authors:** Spencer R. Stinson, Jane K. La Du, Dante M. Perone, Sibel I. Karchner, Neelakanteswar Aluru, Lisa Truong, Mark E. Hahn, Robyn L. Tanguay

**Affiliations:** 1 Sinnhuber Aquatic Research Laboratory, Department of Environmental and Molecular Toxicology, Oregon State University, Corvallis, OR 97331, USA; stinsosp@oregonstate.edu (S.R.S.); jane.ladu@oregonstate.edu (J.K.L.D.); dante.perone@oregonstate.edu (D.M.P.); lisa.truong@oregonstate.edu (L.T.); 2 Biology Department, Woods Hole Oceanographic Institution, Woods Hole, MA 02543, USA; skarchner@whoi.edu (S.I.K.); naluru@whoi.edu (N.A.); mhahn@whoi.edu (M.E.H.)

**Keywords:** aryl hydrocarbon receptor, aryl hydrocarbon receptor interacting protein, zebrafish, development, transcriptomics, hematopoiesis

## Abstract

The Aryl Hydrocarbon Receptor (AHR) is a versatile receptor that binds various compounds, triggering downstream transcriptional changes involved in the regulation of xenobiotic metabolism and cell fate. The Ahr Interacting Protein (AIP), also known as XAP2 and ARA9, is a molecular chaperone essential for the cytosolic retention and stabilization of the AHR. Past studies, and AIP-linked human pathologies, suggest an alternative developmental role of the AIP apart from the AHR and canonical xenobiotic response. A null mutation of the AIP in zebrafish larvae (designated as *aip^wh86^*) resulted in craniofacial malformations, failure to inflate the swim bladder, and an enlarged and darkened liver, emerging at 5–6 days post-fertilization (dpf). Histopathological analysis at 5 dpf suggested no histological differences in the homozygous (HMZ) mutant compared to the wild type (WT). Whole-larvae RNA sequencing at 6 and 7 dpf showed significant dysregulation of key transcripts in the mutant larvae compared to WT, with pathway analysis revealing alterations in embryonic hematopoiesis and cardiac development. RT-qPCR validation showed that transcripts involved in the Hif-mediated pathway were overexpressed as early as 5 dpf. O-dianisidine staining at 6 dpf showed an increase in hemoglobin within the liver and cardiac regions of the mutant AIP larvae. This study demonstrates the complex and diverse role of AIP in vertebrate embryonic development and physiology.

## 1. Introduction

The Aryl Hydrocarbon Receptor (AHR) is best known as a transcription factor involved in adaptive responses to xenobiotic insult; however, it also binds to endogenous molecules. In both cases, the AHR forms a complex with co-chaperone proteins such as the AIP, P23, and two HSP90 proteins. Once bound to a ligand, the complex is transported to the nucleus, dissociates, and then AHR heterodimerizes with ARNT/HIF1β and binds to xenobiotic response elements (XREs). This often leads to downstream activation of cytochrome P450 (CYPs) genes such as CYP1A and other xenobiotic response genes [1].

The AHR is not just known for xenobiotic response but also has endogenous roles in vertebrate development, including maintaining stem cell populations, enhancing tissue repair, immune system development, and organogenesis [2,3]. Without co-chaperones, such as the AIP, the AHR complex is hypothesized to have altered stability, functionality, and ligand specificity [4]. Due to this, it is vital to characterize the co-chaperones of the AHR complex in the context of xenobiotic response, vertebrate physiology, and development. A better understanding of the AHR and its binding partners allows for enhanced mechanistic insight, predictive modeling, and downstream risk assessment to characterize the toxicity of xenobiotic compounds.

The co-chaperone AIP is a 37 kDa protein containing three tetratricopeptide repeat (TPR) domains and seven α-helices. AIP is critical for stabilizing protein complexes and ensuring the proper structural conformation and function of binding partners [5,6]. The third TPR domain is necessary to bind both AHR and hsp90, while the α-helix at the end is necessary to bind AHR [6]. Another structural characteristic is its PPIase-like domain, which is structurally similar to that of the immunophilin protein family. However, because AIP’s PPIase-like domain lacks catalytic activity and is subsequently unable to bind the immunosuppressants FK506 and cyclosporin, it is not a true immunophilin [6]. In the absence of AIP, AhR displays increased degradation and increased nuclear translocation in a ligand-independent manner [7]. The interaction between the AIP and AHR, and how this interaction is balanced between stability and cytosolic retention, is not well understood. Further investigation has shown AIP to interact with a wide range of proteins and receptor complexes that contribute to normal development and physiology. This includes the Hepatitis B virus X protein (HBx), Epstein-Barr virus nuclear antigen 3 (EBNA-3), HSP90, HSC70, phosphodiesterases, estrogen receptor α, glucocorticoid receptors, PPARα, thyroid hormone receptors, EGFR, survivin, and TNNI3K [4,6,8,9]. AIP is a proposed tumor suppressor gene, and cytoplasmic expression in epithelial and stromal pancreatic carcinoma cells is associated with a lower survival rate compared to nuclear expression [10,11].

**Figure 1:**
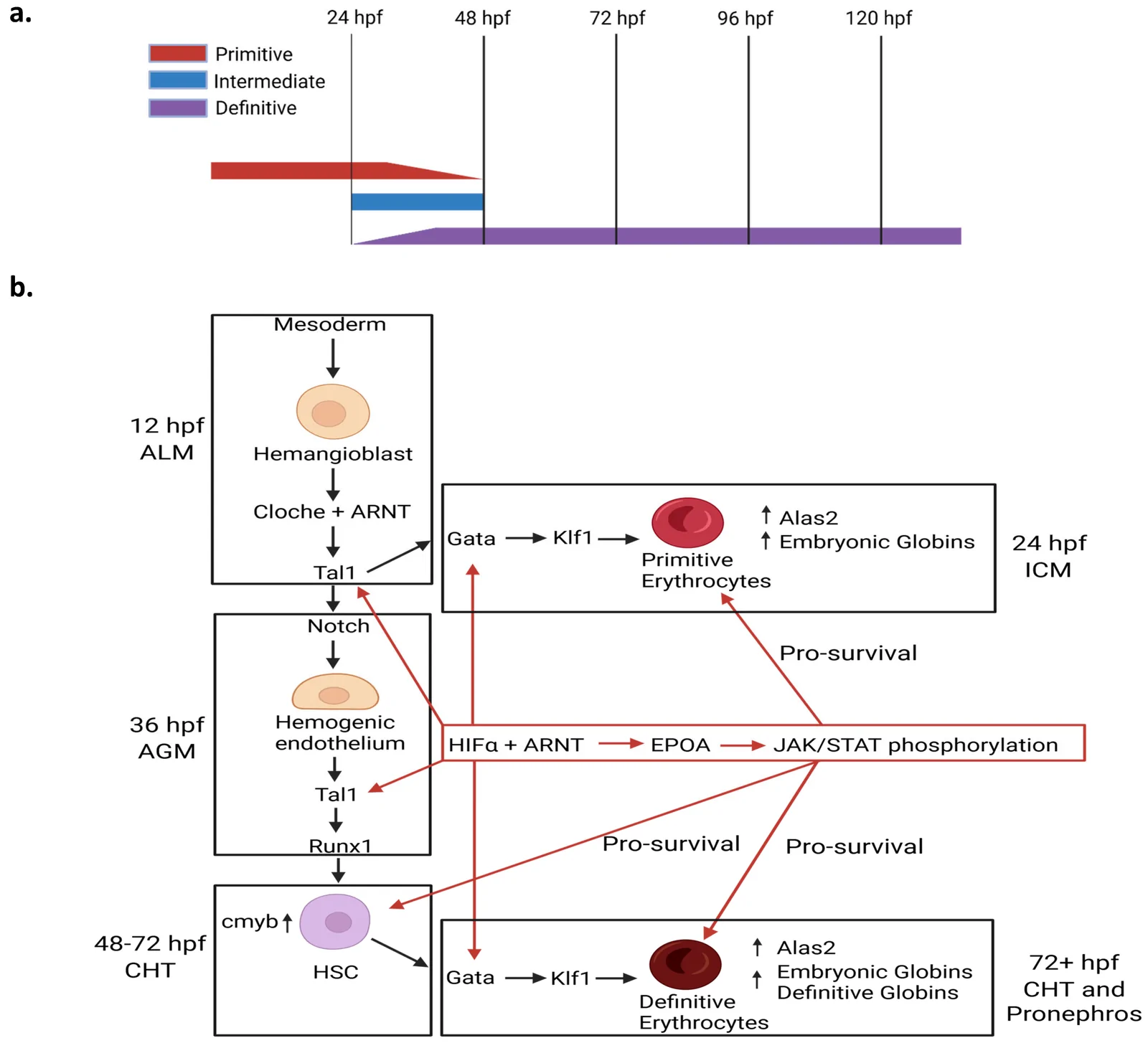
Primitive hematopoiesis begins at embryogenesis, continuing until 1–2 dpf. (**a**) Primitive hematopoiesis tapers off, with primitive cell synthesis gradually decreasing as the definitive phase begins. Primitive erythrocytes are diluted out by definitive cells at ~7 dpf. (**b**) Black boxes and arrows denote the standard hematopoietic pathway throughout embryonic and early larval development in zebrafish. The red box and associated arrows indicate Hif-mediated regulation at multiple steps and time points within the pathway. The process begins with the mesoderm-to-hemangioblast differentiation at the anterior lateral mesoderm (ALM), with primitive hematopoiesis occurring in the intermediate cell mass (ICM), then the aorta gonad mesonephros region (AGM) housing the migrating hemogenic endothelium, followed by hematopoietic stem cell (HSC) differentiation in the caudal hematopoietic tissue (CHT), and definitive hematopoiesis continuing in the CHT and pronephros.

Within adult humans, AIP is expressed in the pituitary and thymus, with lower expression in the liver, kidney, and lung [5,12]. Expression patterns of the AIP in embryonic development is not well characterized, but germline mutations have been linked to complex pathophysiology in humans, such as an increased risk of developing Familial Isolated Pituitary Adenomas (FIPA) and, more recently, lysosomal storage disease [6,13,14,15,16,17,18]. Analysis of heterozygous (HET) *Aip* mutant mice with pituitary tumors shows a loss of wild type *Aip* within the tumor and increased Hif1-α signaling [19]. Reduced differentiation of erythrocyte populations was also observed in *Aip* null mutant mice, demonstrating a potential role for *Aip* in erythrocyte differentiation and potentially hematopoiesis as a whole. However, a potential mechanism is unknown [20]. Hematopoiesis is divided into primitive and definitive stages (Figure 1a), with temporally distinct induction and repression of genes associated with primitive and definitive erythrocytes (Figure 1b). However, this process not only regulates blood cell differentiation and localization but also molecular pathways responsible for heme production and globin switching in development [21,22,23,24], potentially leading to complex blood pathologies if dysregulated [25,26]. The Hypoxia Inducible Factor alpha (HIFα) pathway, which is also highly conserved in vertebrates, is associated with the induction of erythropoiesis in a hypoxic environment and in cancers. Notably, the HIFα pathway also relates to the AHR complex in that HIFα subunits are bHLH-PAS proteins and require heterodimerization with the same binding partner, HIFβ/ARNT, in the nucleus [27,28,29,30,31,32].

Zebrafish are ideal model organisms for studying early developmental toxicity. Their transparent embryos, ex utero development, and small size make them a high-throughput and cost-effective model organism [33]. This also makes the zebrafish an ideal organism to genetically modify, as a large clutch can be microinjected at the single-cell stage without the need to reimplant the embryo in utero. The entirety of the zebrafish genome has been sequenced; approximately 70% of zebrafish genes have a human orthologue, with nearly 84% of disease-related genes in humans having a zebrafish counterpart [34]. Many of the same genetic drivers that regulate key developmental processes, such as axis formation, neural tube formation, organogenesis, somitogenesis, and hematopoiesis, are shared between zebrafish and humans [35,36,37,38,39]. While most mammals have a single copy of the *ahr* gene, zebrafish have 3 orthologs, *ahr1a*, *ahr1b*, and *ahr2*, due to multiple gene duplication events [40]. Among the three, *ahr2* appears to share the most similar function to the human AHR, with respect to toxicological response. This conservation is similarly shown in the zebrafish Aip, where nearly 80% of amino acids are shared between humans and zebrafish. Two *aip*-mutant lines were generated, and both result in early stop codons; the *aip^wh86^* line has an earlier deletion in exon 2, compared to a deletion in exon 5 for the *aip^wh239^* line [41]. For the former, the transcript encodes a protein 86 amino acids in length, lacking all three TPR domains and the α-7 helix. While previous studies utilized both lines to investigate how the zebrafish Aip affects sensitivity to dioxin-like compounds and polycyclic aromatic hydrocarbons (PAHs) [41,42], this study focused on the developmental impacts observed specifically in the *aip^wh86^* line, and potential mechanisms of early lethality.

To better understand the physiological and developmental role of the AIP, this study aimed to phenotypically and transcriptionally characterize the effects of the loss-of-function allele *aip^wh86^* in the developing zebrafish. The downstream applications of elucidating the AIP’s developmental role include a better understanding of AHR signaling, xenobiotic response and physiology, and AIP-related pathologies associated with other molecular pathways.

## 2. Materials and Methods

### 2.1. aip^wh86^ Line Generation

The *aip^wh86^* line has a 2 bp CRISPR–Cas9-induced deletion in exon two of the gene, resulting in a frameshift mutation and early stop codon. *aip* mutants were generated in Tüpfel long fin (TL) zebrafish at Woods Hole Oceanographic Institution with single guide RNA (sgRNA) generation and microinjection protocol previously described [41]. TL larvae were shipped to Oregon State University, where they were crossed with WT AB zebrafish. These larvae were then continuously out-crossed with WT AB fish for multiple generations, establishing a line with primarily an AB background, which were the fish used in this study.

### 2.2. Husbandry and Genotyping

Adult Zebrafish (*Danio rerio*) were housed at the Oregon State University Sinnhuber Aquatic Research Laboratory (SARL) under the guidance of the Institutional Animal Care and Use Committee protocol 2024-0485 and 2024-0510. Adult heterozygous *aip^wh86^* fish were kept in 2.8–6 L tanks with a density of 7 fish per liter in a 14:10 light:dark cycle. Each tank was fed twice a day with Sparos Zebrafeed (300 microns) and connected to a recirculating water system that replenishes tanks with water run through a 10-micron filter and treated with activated charcoal and UV sterilization. The water was supplemented with Instant Ocean and sodium bicarbonate to reach a desirable pH of 7.4. Heterozygous adult *aip^wh86^* fish were placed in spawning baskets with males and females separated by a gate. At approximately 08:00 fish room lights were turned on and the gates were removed to initiate spawning. At 09:00, embryos were collected and placed in glass petri dishes in embryo medium (EM) consisting of filtered water with 15 mM NaCl, 0.5 mM KCl, 1 mM MgSO_4_, 0.15 mM KH_2_PO_4_, 0.05 mM Na_2_HPO_4_, and 0.7 mM NaHCO_3_ [43]. Unfertilized or necrotic embryos were removed before being counted and placed in 150 mm petri dishes at a density of ~100 embryos per dish. At 4 dpf, larvae were genotyped while alive with the Zebrafish Embryonic Genotyper (ZEG) (wFluidX, Inc., Salt Lake City, UT, USA). Individual larvae were collected into 12 µL droplets of EM and placed onto the ZEG slide. The slide has a sticky film that prevents water droplets from sliding across it during vibration and contains 24 slots for 24 individual larvae. The slide was inserted into the ZEG instrument and ran at 11 V for 7.5 min. The ZEG instrument vibrates the larvae, shedding excess cells into the surrounding EM droplet while maintaining their integrity. The larvae were then replated, and the droplets were moved into a separate plate for genotyping. Careful labeling was necessary to ensure the water droplets could be associated with the proper individual larvae for downstream genotype labeling. A TaqMan probe associated with two dyes, VIC for WT and FAM for mutant alleles, was used to genotype the larvae (Supplementary Table S1). 2.5 µL 2× TaqMan Enzyme, 0.25 µL 20× TaqMan Assay solution, and 1.5 µL ultra-pure (UP) H_2_O were used per reaction for a total of 4.25 µL. The master mix was added to a 384-well plate, followed by the addition of 0.75 µL of genomic DNA (gDNA) collected via ZEG. The plate was spun at 4000 rpm for 4 min, then inserted into a QuantStudio 5 plate reader instrument (ThermoFisher, Waltham, MA, USA). From here, if larvae are labeled as *aip^wh86^*, they are considered HMZ mutants, unless otherwise specified. Wild type larvae will be labeled as WT.

### 2.3. Histological Preparation

Agarose blocks were prepared by melting fresh 1% agarose in ddH2O, then pouring it into molds lined with tape. The mold was 5 mm in height, and 8 mm of tape was added to create a total agarose height of 3 mm. The height of tape can be adjusted depending on how tall the block needs to be. The mold was then placed at 4 °C until hardened, the tape was then removed, and the block was separated. This created an agarose block with distinct lanes, allowing for easier positioning of larvae. Around 15 larvae were euthanized in ice-cold tricaine (160 mg/L) and rinsed twice in 10% neutral buffered formalin (NBF) before being pooled, based on genotype, in 15 mL conical tubes. 5 mL of 10% NBF was added to the conical tubes, and parafilm was used to seal the tops of the tubes before incubating overnight at 4 °C. The next day, 5 mL of 70% ethanol was added to two new 15 mL conical tubes, and the larvae were transferred into them before incubation at room temp (RT) overnight. Larvae were then pipetted out of their respective tubes into separate agarose blocks. WT and *aip^wh86^* larvae were positioned using a micro-point round tool to align them in their respective lanes, ensuring they were all within the same position (*n* = 13–16) (Supplementary Figure S1a). Both sagittal and coronal alignment was performed. 1% agarose was prepared, kept on a hot plate at 60–65 °C, and pipetted onto the larvae after positioning. Extra caution was taken to ensure that larvae were not dislodged from their positions by the addition of the agarose. The mold then sat for 1 min before being placed in an embedding cassette and submerged in 10% NBF, where they were stable until paraffin embedding and sectioning. Subsequent paraffin embedding, sectioning (10-micron cuts), and H&E staining were performed at the Oregon State University Veterinary Diagnostic Laboratory (VDL). After staining, blinded samples were sent to an expert fish pathologist for analysis. Cardiac, liver, and swim bladder regions were scored for histological abnormalities using a one-tailed *t*-test.

### 2.4. RNA Extraction

Once genotyped, larvae were moved into four separate petri dishes—two for WT and two for *aip^wh86^*. The heterozygous larvae were excluded from the study. RNA was extracted at 6 and 7 dpf to account for the progressive morphological phenotypes. At both timepoints, one petri dish from each genotype, with approximately 100 larvae, was selected and placed on ice. After euthanasia, larvae were added to a 96 well plate, with 1 larvae per well. Nearly all of the EM was removed from the 96-well plate and replaced with a solution containing 45 µL of EM and 45 µL of 2X RNA Shield (Zymo Research, Irvine, CA, USA) for 30 min at room temperature. Using a P1000 pipette, 100 µL was pipetted into a 96-well homogenization plate containing 400 µL of 1 mm zirconium silicate beads and 500 µL of Zymo Lysis Buffer, from a Zymo Quick-RNA™ MagBead RNA Extraction kit (Cat: R2133). Another 100 µL of Zymo Lysis Buffer was added after all larvae had been plated in the homogenization plate. The plate was sealed with a silicone seal and homogenized with a Mini-Beadbeater-96 (BioSpec Products, Inc., Bartlesville, OK, USA) for 1 min and 15 s at 2400 rpm. The plates were centrifuged for 5 min at 3000 *g*. A liquidator was used to transfer 550 µL of supernatant into two deep-well 96-well plates. 275 µL of ethanol and 30 µL of MagBeads were added to the plates, followed by an automated extraction protocol on the KingFisher instrument (ThermoFisher, Waltham, MA, USA), following a Zymo MagBead RNA extraction protocol. This involved bead incubation for 10 min, followed by two wash steps in 500 µL of DNA/RNA wash buffer for 2 min, and a final wash in 500 µL of ethanol for 2 min. Lastly, beads were incubated in 50 µL of DNase, followed by a 10-min incubation in 500 µL of RNA prep buffer. The beads were then washed in 500 µL of ethanol for 2 min, followed by 10 min of air drying, and elution in 50 µL of molecular-grade water. Sample quality control (QC) was performed on each extraction plate using an Agilent 4150 TapeStation System (Agilent, Santa Clara, CA, USA). Samples with RIN scores ≥ 9, indicating high-quality RNA, were pooled by genotype, with 5 larvae per sample, for a total of 4 samples per genotype per day. A Zymo RNA Concentrator Kit (Irvine, CA, USA, Cat No. R1018) was used to further concentrate the RNA samples. A Qubit fluorometer (Invitrogen, Waltham, MA, USA) was used to determine concentrations of pooled samples before sequence submission. All prior steps were repeated for 7 dpf. Samples were randomly selected and pooled based on genotype for both timepoints.

### 2.5. RNA Sequencing and Analysis

RNA samples were placed on dry ice and shipped to Lexogen, Inc., Vienna, Austria, for sequencing. CORALL full-length RNA Sequencing was performed with a depth of 20 million reads per sample. Lexogen provided initial bioinformatic analysis, with adapter, primer, and poly-A tail trimming with the cutadapt tool [44]. The STAR-aligner tool [45] was used to map sequences to the GRCz11 zebrafish genome assembly, and read counts were generated with HTSeq [46]. Both days 6 and 7 had 5 samples for WT and *aip^wh86^*, with pools of 5 larvae per sample. A WT and an *aip^wh86^* outlier were detected and subsequently removed for the day 6 analysis, leaving 4 samples for each condition on day 6 and 5 on day 7, after QC. Principal component analysis (PCA) was performed to analyze relative clustering of groups (Supplementary Figure S2). Raw read counts were first uploaded to R-studio, and transcripts with 0 expression across all samples were filtered out of the analysis. Ensembl transcript IDs (ENSDART) were then collapsed to gene-level counts, ensuring alternative transcript isoforms were identified as a single gene for analysis. The DESeq2 R-package [47] was used to perform subsequent differential gene expression and statistical analyses between WT and *aip^wh86^* larvae. Using the Biomart R-package [48,49], Ensembl transcript IDs were mapped to gene names, and transcripts not labeled as protein-coding were further filtered. A log_2_-fold change (log2FC) cutoff of |1| and an adjusted *p*-value cutoff of <0.05 were used for downstream differentially expressed gene (DEG) analysis. DEGs were converted into a downloadable Excel document, and EntrezIDs were uploaded into Cytoscape (Version 3.10.3) for gene ontology and functional analysis (Supplementary Data File). The Cytoscape (Version 3.10.3) ClueGO package (Version 2.5.10) [50] was used to determine clustering and network formation with the “Functional Analysis” configuration selected. GO:BP terms were selected, and a Bonferroni step-down correction method was used to derive corrected *p*-values. The network specificity and kappa score connectivity was set at medium, such that node edges were connected if greater than or equal to a k-value of 0.05. Nodes had a significance threshold of <0.05. Terms sharing more than 50%, were merged into a single term with the highest significance. The prefuse force-directed layout was used for the ontological map, with node size corresponding to greater significance. Transcripts that had no GO annotation compared to the most recent GO Biological Process EBI UniProt database [51] were automatically filtered out during the ontological mapping of the transcripts. Additionally, G:Profiler [52] was used to detect more specific GO terms. While ClueGO represents broader terms and sacrifices specificity to form a network and increase simplicity, G:Profiler presents specific terms, with no attempt at explicit connection. Both are good at identifying GO terms, but they differ in how they present them and how they are interpreted.

### 2.6. RT-qPCR

Primers were designed using NCBI Primer BLAST (Primer3, version 2.5.0) [53] against the Zebrafish genome for RNA transcripts of interest. The 11 target transcripts were *embryonic hemoglobin alpha 1* (*hbae1*), *embryonic hemoglobin beta 1* (*hbbe1*), *matrix metallopeptidase 9* (*mmp9*), *egl-9 family hypoxia inducible factor 2* (*egln2*), *kruppel like factor 1* (*klf1*), *aminolevulinate delta-synthase 2* (*alas2*), *erythropoietin a* (*epoa*), *gata binding protein 1* (*gata1*), *t-cell acute lymphocytic leukemia 1* (*tal1*), *myb proto-oncogene* (*cmyb*), *RUNX family transcription factor 1* (*runx1*), and *beta-actin* (β-*actin*) (Supplementary Table S2). Primer validation were performed using WT 48-h post-fertilization (hpf) cDNA in standard PCR, using a KOD Hot Start Polymerase (Millipore Sigma, Burlington, MA, USA, SKU: 71086-5) (Supplementary Figure S3). The ThermoFisher (Waltham, MA, USA) RNA-to-CT 1-Step kit was used with an input of 20 ng RNA for all genes except for *epoa*, which had an input of 40 ng, due to its low basal expression in WT larvae at 6 dpf. QuantStudio 5 was used to run the RT-qPCR assay, and the delta delta Ct method was used to derive fold change values relative to a *β-actin* housekeeping control. A log2FC transformation was performed to get relative increases in transcript expression relative to WT. 1-tailed *t*-tests were performed for each gene of interest at each time point based on delta Ct values. A one-way ANOVA was also performed to determine whether gene expression changes were significant across timepoints for the *aip^wh86^* larvae, with post hoc *t*-tests conducted on significant timepoints to identify specific gene and time differences.

### 2.7. O-Dianisidine Stain and DMOG Exposure

We used O-dianisidine staining to investigate hemoglobin production in *aip* mutants. The stock solution prepared was 1.4 mg/mL of O-dianisidine powder (ThermoFisher, Waltham, MA, USA, CAS: 119-90-4) dissolved overnight in 100% Ethanol. A modified version of [54] was used for O-dianisidine staining. A working stock of 2 mL of stock, 500 µL of 0.1 M NaOAc (pH ~4.5), 2 mL ddH_2_O, and 100 µL 30% H_2_O_2_ was prepared. Two rounds of larvae, the first with 5 and the second with 4, were placed in each well of the top row of a 24-well plate with 500 µL of working solution. The larvae were stained for 30 min in the dark at RT, then washed 3x in ddH_2_O. Larvae were fixed overnight in 4% paraformaldehyde (PFA) at 4 C in the dark. After fixation, the larvae were washed 2x in PBS. Immediately after washing, the larvae were imaged under an Olympus SZ51 stereo microscope at 30× magnification. The light of the stereo microscope was adjusted to create the greatest contrast in O-dianisidine staining compared to larval background tissue. Once a control embryo was selected, the light was unadjusted for the rest of the images taken for consistent imaging. Images from both experimental groups from different days/rounds were combined for the analysis for a total *n* = 9.

For a positive control of *hif* activation and staining, larvae were exposed to the Phd1 inhibitor, Dimethyloxallyl Glycine (DMOG) (Millipore Sigma, Burlington, MA, USA, CAS: 89464-63-1). A total of 11 larvae were exposed to 150 µM DMOG from 4–6 dpf, with imaging at 6 dpf (Supplementary Figure S4). Larvae were exposed in petri dishes following a daily renewal of chemical media. At 6 dpf, larvae were washed 3x in ddH_2_0 and stained following the same protocol described above. After imaging, a fixed region of interest was created in the shape of a rectangle encompassing the liver and cardiac region of the larvae through ImageJ/Fiji. The shape was saved in the Region of Interest (ROI) manager to ensure a consistent area when measuring the pixel intensity of the stain. The derived pixel intensity was averaged and analyzed using a 1-tailed *t*-test (*n* = 9 per group) to derive a significance value. Averaged values were plotted as a box and whisker plot in Microsoft Excel.

### 2.8. hif-2α Translation Inhibitor Exposure

Our exposure protocol was modified from [55]. A 100 mM stock solution of *hif*-2α Translation Inhibitor, methyl 3-2[2-[cyano(methylsulfonyl)methylene]hydrazino]thiopene-2-carboxylate (Compound 76) [55,56] was made by dissolving pure powder (Millipore Sigma, Burlington, MA, USA, 400087, CAS: 882258-69-1) in anhydrous DMSO. Heterozygous *aip^wh86^* fish were crossed, resulting in a Mendelian ratio of 25% HMZ, 25% WT, and 50% HET. Clutches were separated into two Petri dishes with 200 larvae per dish. One dish was exposed to a 1% DMSO control solution in embryo medium, while the other was exposed to 10 µM compound 76. A fresh compound 76 solution was made each day, and the exposure medium was renewed. The exposure started at 4 dpf with a planned end at 9 dpf, with lethality and liver phenotypes counted for each plate. The tracking stopped early at 7 dpf when early lethality and liver phenotypes remained unchanged in exposed *aip^wh86^* larvae. A subset of larvae were euthanized at 6 dpf for RNA extraction and subsequent RT-qPCR analysis of Hif and hematopoietic-related gene transcripts.

## 3. Results

### 3.1. aip^wh86^ Larvae Exhibit Morphological Abnormalities

**Figure 2:**
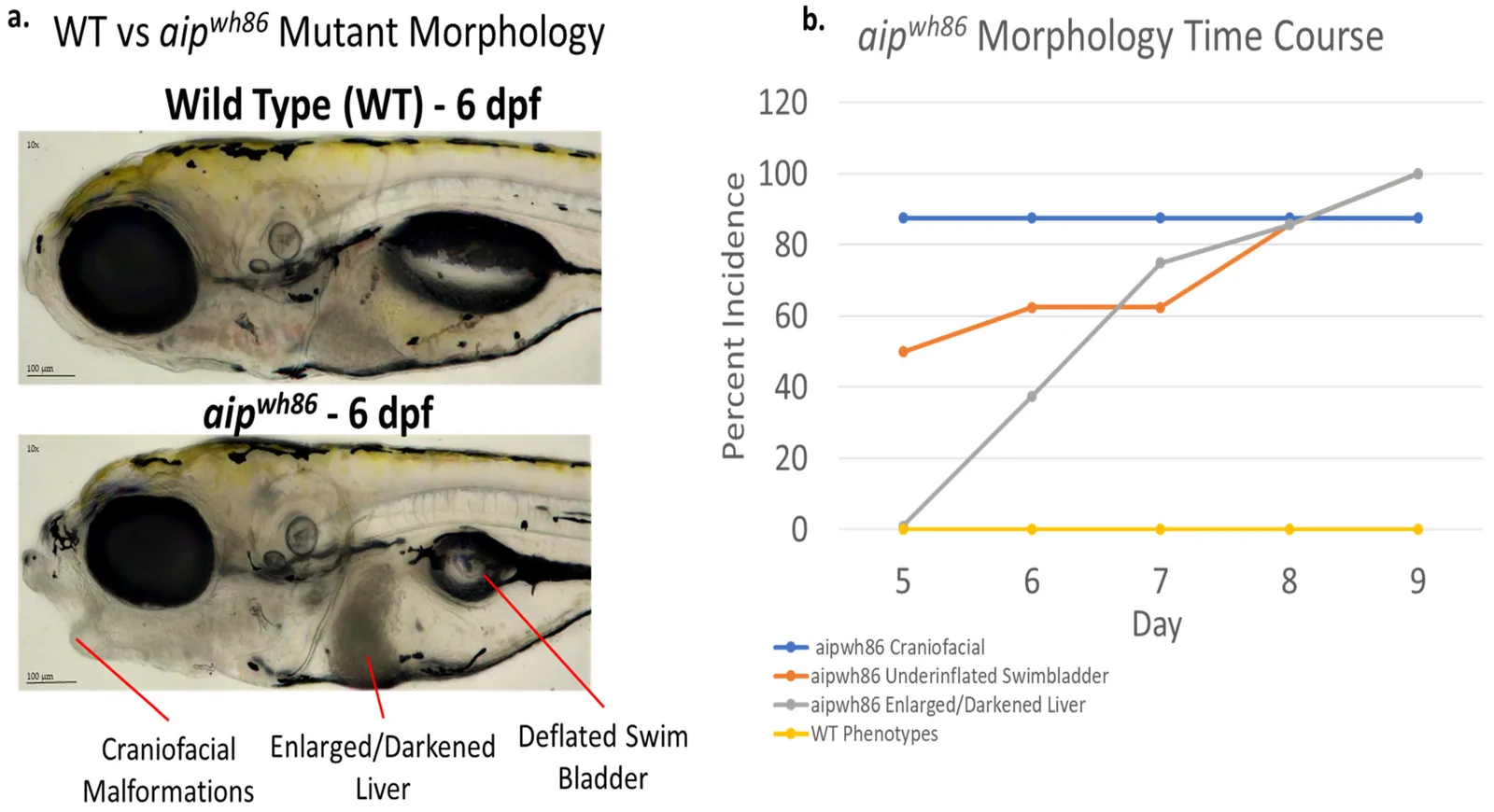
(**a**) Morphological comparison of 6 dpf wild type larvae versus *aip^wh86^* larvae. (**b**) Quantified time-course imaging of mutant morphological abnormalities starting at 5 dpf until mortality (*n* = 8). *aip^wh86^* phenotypes emerged at 5 dpf, beginning with partial deflation or lack of inflation of the swim bladder. Before this, the *aip^wh86^* larvae are indistinguishable from WT siblings. Craniofacial abnormalities are also observable at 5 dpf. Liver enlargement and darkening are first observed at 6 dpf, progressing until early lethality at 9 dpf. WT siblings displayed none of the same morphological abnormalities during the same time course.

Craniofacial abnormalities were present in most larvae at 5 dpf (Figure 2a), and the incidence remained constant through 9 dpf (Figure 2b). The *aip^wh86^* larvae either showed no inflation of the swim bladder or failed to maintain inflation over the time course, starting at 5 dpf. The consistent and definitive emerging phenotype that began at 6 dpf was a significant increase in liver size and discoloration. From 6 to 9 dpf, both abnormal swim bladder and liver incidence increased among the *aip^wh86^* larvae, with WT having no detectable abnormalities throughout the time course (Figure 2b). By 9–10 dpf, mutant larvae exhibited lethality. Despite morphological abnormalities emerging at 5 dpf, mutant larvae exhibited no observable histological changes at 5 dpf compared to WT siblings (Supplementary Figure S1b). The sections provided adequate tissue quality to analyze the swim bladder, heart, and liver, but prevented confident histological analysis of craniofacial tissues. ImageJ/Fiji (Version 1.54p) analysis of the liver showed increased total size in *aip^wh86^* larvae at 6, 7, and 8 dpf (Supplementary Figure S5).

### 3.2. Whole-Larvae RNA Sequencing Reveals Transcriptomic Differences Between WT and aip^wh86^ Larvae

**Figure 3:**
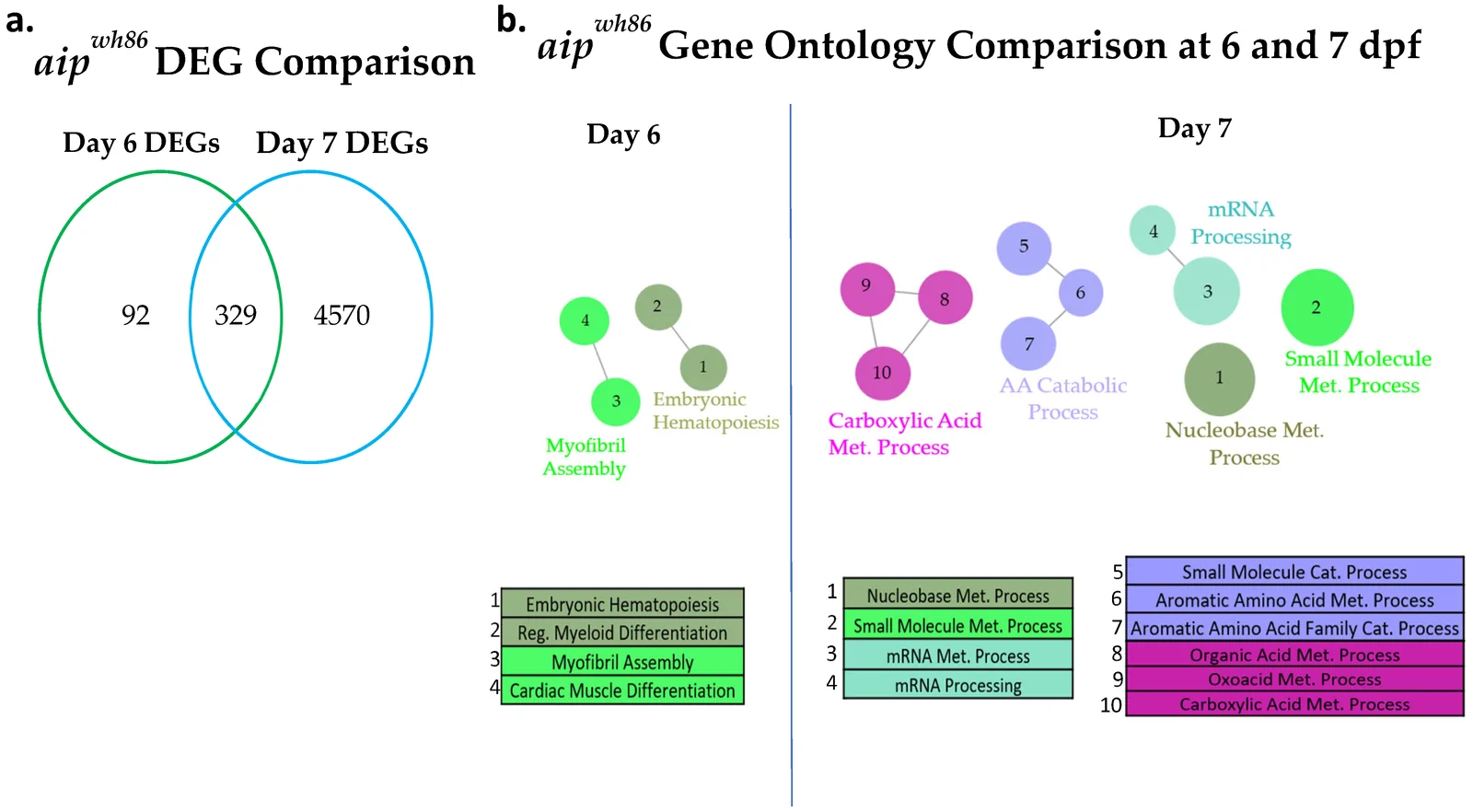
Lexogen CORALL full-length RNA sequencing of both AIP mutant and WT zebrafish larvae was performed at 6 and 7 dpf. The DESeq2 R-studio package was used for differential gene expression analysis, with Log2FC threshold of >1 and *p*-value < 0.05. (**a**) A venn diagram comparing DEGs detected at day 6 and day 7 shows the number of shared DEGs and the number of unique DEGs between days. (**b**) Total DEGs for both days were separately input into the Cytoscape CLUEGO package for gene ontology analysis. Day 6 showed specific dysregulation in genes associated with embryonic hemopoiesis and myofibril assembly/cardiac development. Day 7 showed broader transcriptional changes in pathways associated with physiological metabolic processes, such as amino acid, nucleobase, carboxylic acid, small molecule, and mRNA metabolism.

**Figure 4:**
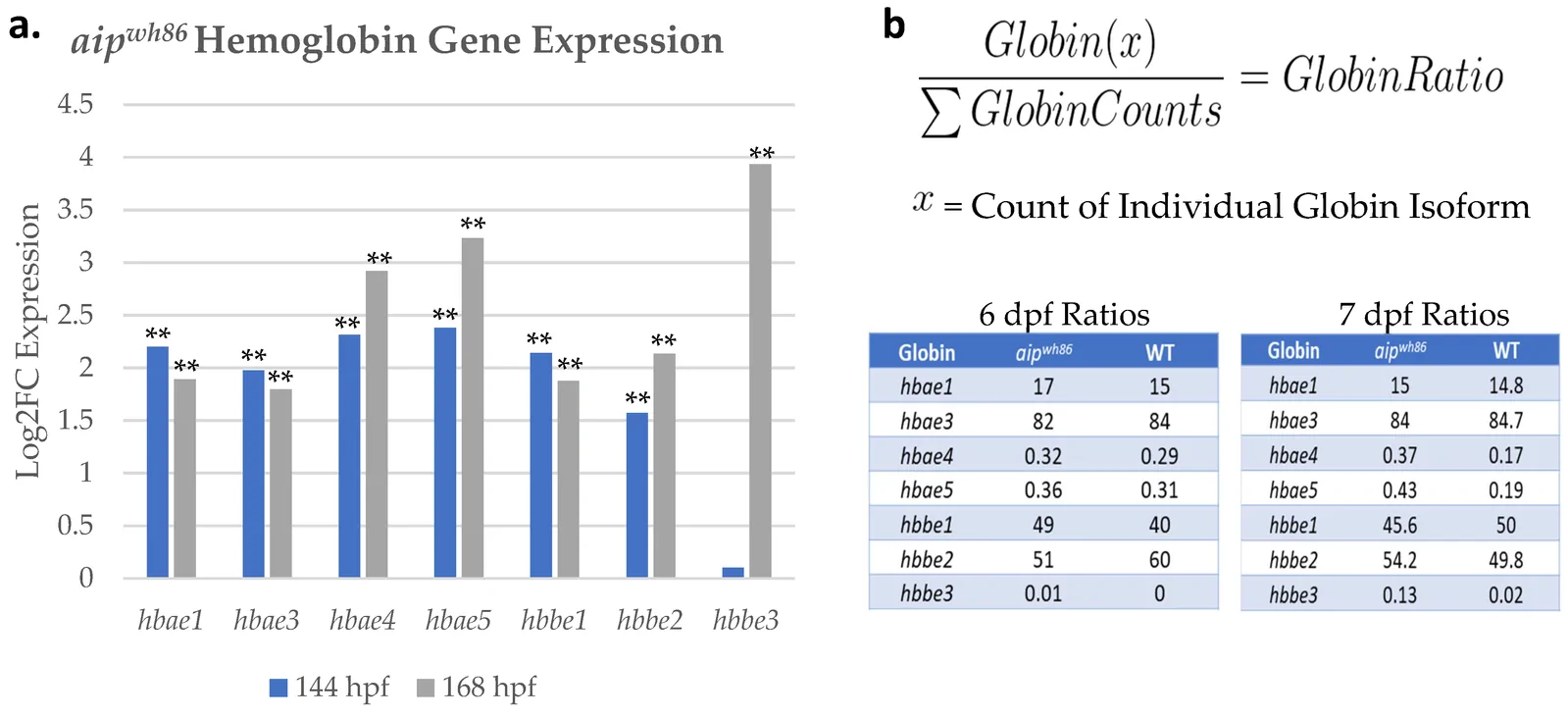
(**a**) Changes in embryonic globin expression (log2FC) at 6 and 7 dpf based on RNA sequencing counts (adj *p*-val < 0.01 **). At both time points, there is prolonged overexpression of hbae1 and 3, and hbbe1 and hbbe2. (**b**) Ratios of globin isoforms were calculated by averaging globin raw transcript counts across samples and dividing by the sum of the globin count averages; a representative equation is shown. This was done separately for alpha and beta globins. The ratio of embryonic globins at day 6 and day 7 is similar between WT and *aip^wh86^* mutant larvae, except for a statistically significant increased ratio of hbae4 and hbbe3 in the mutant larvae at 7 dpf.

Full transcriptomic profiling revealed 421 DEGs in the *aip^wh86^* larvae at 6 dpf using an adjusted *p*-value cutoff of 0.05 and log2FC cutoff of |1|. In contrast, the day 7 larvae contained 4899 DEGs, over 10× more DEGs than the day 6 larvae. The number of differentially expressed genes (DEGs) between days 6 and 7 was compared and represented in a venn diagram, with 92 DEGs unique to day 6, 4570 unique to day 7, and 329 shared between the days (Figure 3a). Gene network analysis of DEGs detected at day 6 revealed pathways associated with embryonic hematopoiesis and myofibril/cardiac development to be affected, and broader metabolic processes affected at day 7 (Figure 3b). Transcripts involved in myofibril assembly and cardiac development, such as *vcp* and *klf1*, are dysregulated in the mutant larvae compared to WT. Transcripts associated with hemoglobin formation were significantly increased, as demonstrated in the RNA sequencing data, where multiple homologs of *hbae* (*1*, *3*, *4*, and *5*) and *hbbe* (*1*, *2*, and *3*) were elevated (Figure 4a). The transcript for *alas2*, responsible for heme synthesis, was also increased. Though embryonic globin expression was increased at day 6 and day 7, the relative ratios of alpha and beta globins were maintained apart from *hbae*4 and *hbbe3* at day 7 (Figure 4b). Ratios were determined by summing the averaged counts of a specific globin, such as *hbae1*, and dividing by the total averaged sum of all alpha globin counts. Other erythroid-associated transcripts, such as *klf1* and *epoa*, were both elevated. Additionally, *socs3a* and *3b*, genes associated with the negative feedback inhibition of the *epoa-jak*/*stat* pathway, were also elevated. Nearly all of the DEGs found at day 6 were shared with day 7, but with a higher magnitude of either decreased or increased expression at day 7. While many of the hematopoietic transcripts remained constant in terms of fold induction, *epoa* and *mmp9* were increased by 1.8× and 1.5×, respectively, at day 7 compared to day 6 (Supplementary Table S3). The Cytoscape network for day 7 revealed more clusters of nodes and a broader range of metabolic processes affected. These nodes included small molecule metabolic processes, carboxylic acid metabolic processes, aromatic amino acid metabolic processes, nucleobase metabolic processes, and mRNA metabolic processes. Within these nodes, carboxylic acid processes, small molecule metabolic processes, nucleobase metabolic processes, and aromatic amino acid metabolic processes had nearly all decreased expression of key transcripts involved in homeostatic endogenous metabolism. Conversely, the cluster associated with mRNA metabolism showed a significant elevation of transcripts associated with homeostatic RNA metabolism.

### 3.3. Expression and Ratios of Embryonic Globin Transcript Abundance Are Altered in aip^wh86^ Larvae

Mutant larvae showed increased abundance of *hbae1*, *3*, *4*, and *5*, and *hbbe1* and *2* at 6 dpf. The same was observed at 7 dpf, with the addition of *hbbe3* overexpression (Figure 4a). At 6 dpf, the embryonic globins *hbae1* and *3*, and *hbbe1* and *2*, showed comparable ratios of total transcript raw read counts in *aip^wh86^* larvae to those of WT (Figure 4b). However, at 7 dpf, *aip^wh86^* larvae showed a statistically significant difference in the overabundant ratio of *hbae4* in mutant larvae compared to WT. Furthermore, in the *aip^wh86^* larvae, *hbae5* is overexpressed at 6 and 7 dpf. At 6 dpf, *hbae4* was overexpressed in *aip^wh86^* larvae, but *hbbe3* was expectedly decreased. Similarly, at 7 dpf, *hbae4* was overexpressed in *aip^wh86^* larvae, but in contrast, mutant larvae showed a spike in *hbbe3* transcript abundance. This transcript decreased on day 6 but saw increased transcription on day 7.

### 3.4. RT-qPCR Shows Hematopoietic Dysregulation at 5 and 6 dpf in aip^wh86^

**Figure 5:**
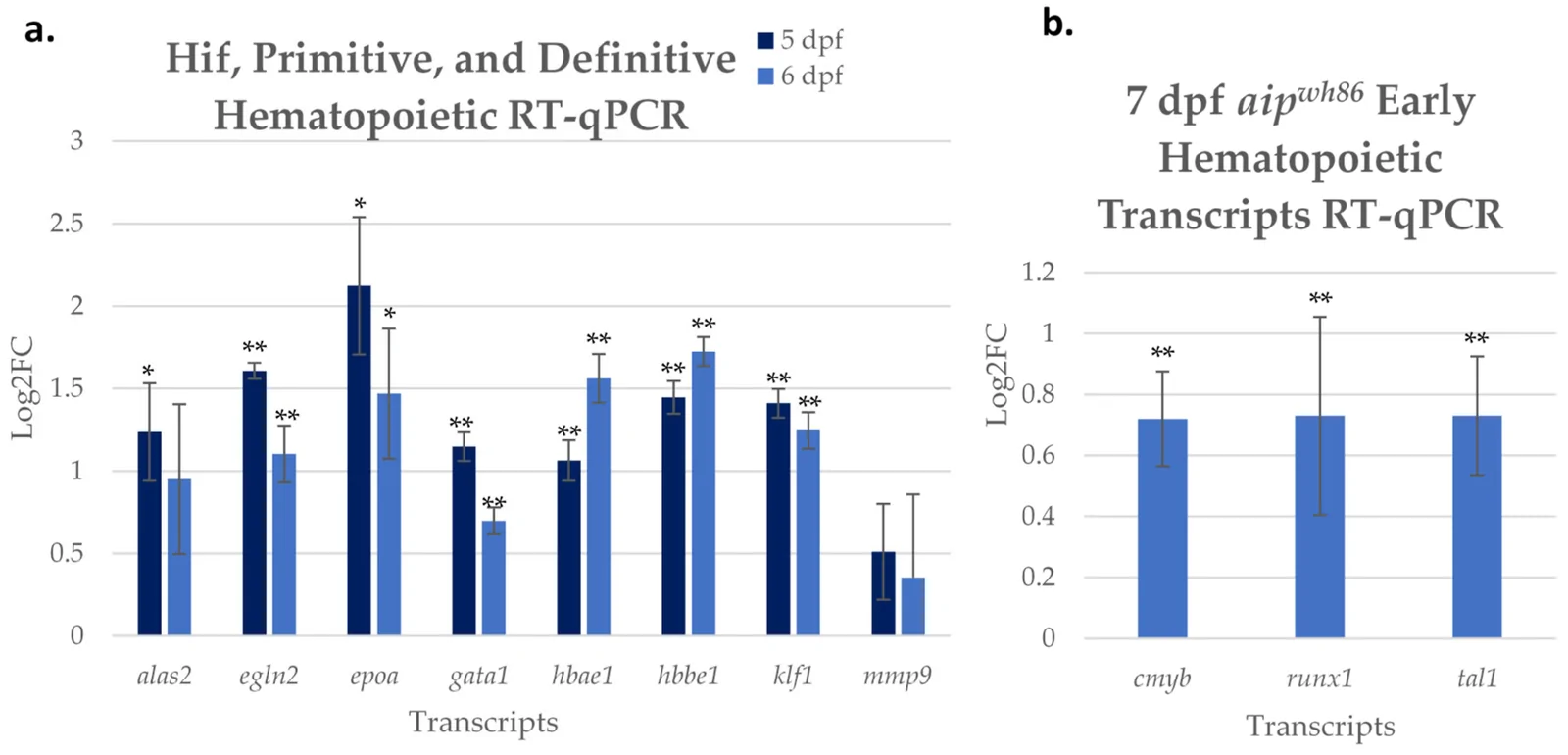
(**a**) RT-qPCR analysis of Hif-related, primitive hematopoietic, and definitive hematopoietic transcripts at 5 and 6 dpf, all normalized to *β-actin* (*p*-val < 0.05 *, *p*-val < 0.01 **, student’s 1-tailed *t*-test, error bars represent SEM). RNA was extracted at 5 and 6 dpf to determine if transcriptional changes of these genes were detectable at an earlier time point. At both timepoints mmp9 displayed no significant changes in expression, while all other transcripts were overexpressed compared to the control. At 6 dpf, all other transcripts apart from *alas2* were significantly increased. Transcriptional changes between 5 and 6 dpf were statistically similar, with *hbae1* being the only transcript significantly different between time points. (**b**) RT-qPCR of early stem-like hematopoietic transcripts associated with progenitor cell differentiation at 6 dpf, normalized to *β-actin* (*p*-val < 0.01 **). All transcripts associated with the differentiation, proliferation, and survival of Hematopoietic Stem Cells (HSCs) were significantly overexpressed in *aip^wh86^* mutants compared to WT controls.

RT-qPCR validation of early hematopoietic and Hif target genes was performed to determine whether increased expression could be detected earlier at the 5 dpf timepoint, compared with 6 dpf. This RT-qPCR panel included 8 genes—*alas2*, *hbae1*, *hbbe1*, *egln2*, *klf1*, *epoa*, *mmp9*, and *gata1*, based on their elevated expression in *aip^wh86^* larvae from the RNA sequencing. Despite not showing differential expression in the RNA sequencing at 6 dpf, the master erythroid transcription factor, *gata1*, was included due to its established role in hematopoiesis. Nearly all transcripts showed robust elevation at both time points, indicating that expression changes occur as early as day 5 and maintain overexpression into day 6 (Figure 5a). *alas2* showed increased expression at 5 dpf and significantly decreased at 6 dpf. In contrast, despite showing increased expression in the RNA sequencing dataset, *mmp9* displayed no statistically significant differential expression at either 5 or 6 dpf by RT-qPCR. All transcriptional changes between timepoints were statistically similar, except *hbae1*, which showed an increase at day 6. A second panel of transcripts was analyzed at 6 dpf through RT-qPCR to further explore earlier pathways of hematopoiesis, including the establishment and maintenance of hematopoietic stem cells (HSCs). This panel included the transcripts *tal1*, *runx1*, and *cmyb*. Though these transcripts were not detected in the RNA sequencing data at 6 or 7 dpf after DESeq2 analysis, RT-qPCR displayed an overexpression at 6 dpf in *aip^wh86^* larvae (Figure 5b).

### 3.5. aip^wh86^ Larvae Exhibit Higher Hemoglobin Content via O-Dianisidine Staining

**Figure 6:**
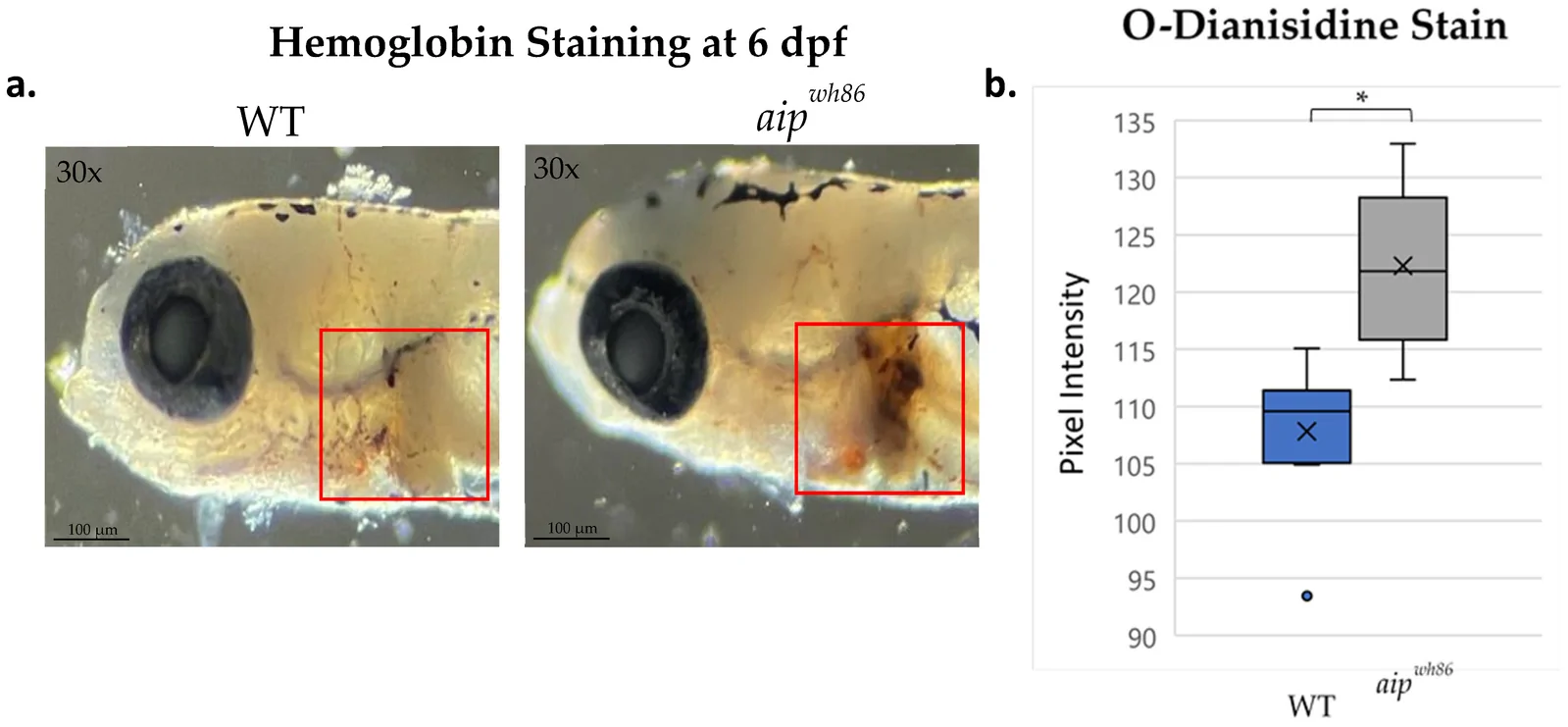
(**a**) Hemoglobin staining with 600 µg/mL of O-dianisidine was performed on WT and *aip^wh86^* larvae (*n* = 9), with a red box denoting the recorded region of interest, imaged at 30×. (**b**) Images were analyzed in ImageJ to quantify the relative stain intensity through pixel analysis, with the x denoting mean stain intensity (*p*-val < 0.05 *). Pixel intensity analysis of the region of interest showed a statistically significant increase in *aip^wh86^* larvae compared to WT (student’s 1-tailed *t*-test).

Based on the transcriptomic profile of the *aip^wh86^* larvae, we hypothesized increased hemoglobin production. To test this experimentally, the mutant larvae were stained using O-dianisidine, which measures the relative amount of hemoglobin based on stain intensity. Mutant larvae at 6 dpf had an increase in stain intensity in the cardiac and hepaticobilliary region of the larvae compared to WT larvae (Figure 6a). This increase was confirmed statistically through image analysis with fixed regions of interest (Figure 6b). Treatment with DMOG, a PHD inhibitor and subsequent Hif-2α activator, was used as a positive control in O-dianisidine staining. Increased hemoglobin staining intensity was observed at day 6 in larvae treated with 150 µM of DMOG from 4–6 dpf.

### 3.6. Compound-76 Treatment Reveals Partial Rescue of Dysregulated Hematopoietic Transcripts

**Figure 7:**
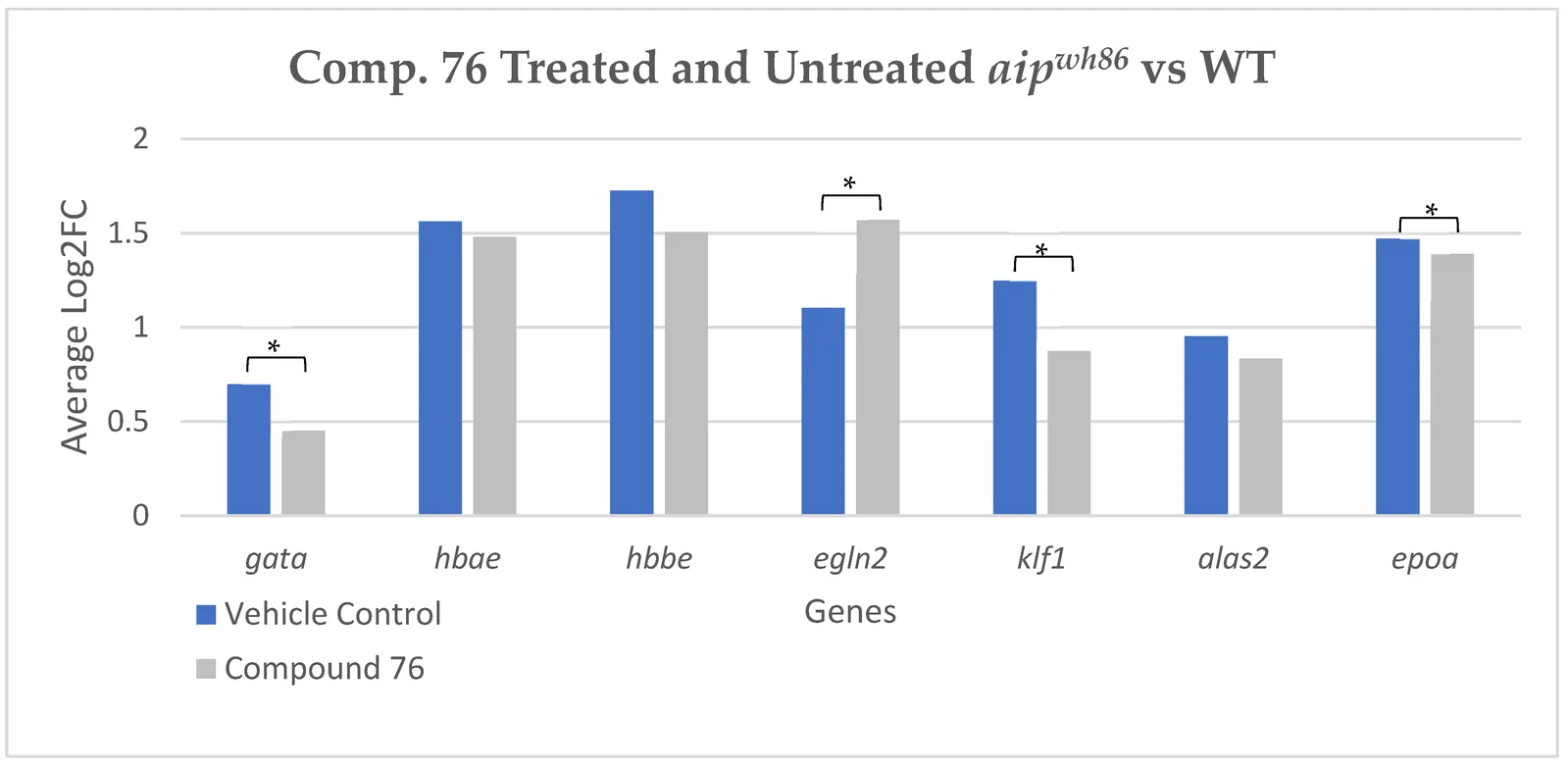
RT-qPCR analysis of HIF-related, primitive hematopoietic, and definitive hematopoietic transcripts comparing *aip^wh86^* larvae to *aip^wh86^* larvae treated with 10 µM compound-76 (*p*-val < 0.05 *). RNA was extracted at 6 dpf, pooled in groups of 5 larvae, and run in triplicate. *aip^wh86^* larvae and compound-76-treated *aip^wh86^* larvae were compared to WT RNA levels and a *β-actin* housekeeping gene using the delta-delta-Ct method. Values represent log_2_FC differences between each group and WT. Multiple *t*-tests displayed a significant decrease in transcript abundance of *gata1*, *klf1*, and *epoa* in the treated mutants compared to untreated, with the abundance of *egln2* transcript increasing after treatment.

Due to increased erythroid and hematopoietic transcripts, along with known crosstalk between Hif-*α* and *ahr* through a shared heterodimerization partner, *hif-α* overactivation was a prime candidate for further analysis. Though Hif-*α* contains two isoforms (Hif-1α and Hif-2α), Hif-2α was primarily selected due to its function as a chronic hematopoietic inducer, rather than an acute inducer like Hif-1α [57]. The *hif-2α* RNA inhibitor, compound-76, was used to assess whether *hif-2α* overactivation was responsible for morphological and transcriptional changes in *aip^wh86^* larvae. Both *aip^wh86^* and WT larvae were treated with 150 µM of the compound and compared to untreated *aip^wh86^* and WT. Pharmacological rescue would be determined by decreased morphological severity or decreased overexpression of Hif-regulated transcripts. Larvae were assessed for morphological rescue by analyzing swim bladder, craniofacial, and liver phenotypes and transcriptional rescue via RT-qPCR of the same Hif and hematopoietic gene panel previously discussed. While treatment of *aip^wh86^* larvae with 10 and 100 µM of the compound yielded no morphological rescue, there was a decrease in the abundance of *gata1*, *klf1*, and *epoa* transcripts, and an increase in the abundance of *egln2*. All other transcripts showed no change in the treated versus untreated *aip^wh86^* (Figure 7).

## 4. Discussion

While the AIP has been linked to protein complexes other than the AHR and to other complex pathologies in humans, its role in development and standard vertebrate physiology is poorly understood [9]. This is especially complex, as many organ systems and physiological changes are occurring during the 5–9 dpf timepoint of the larval zebrafish, along with cell-specific expression of the *aip*. Adjacent analysis of *aip^wh86^* and WT transcripts reveals that maternal WT transcripts are maintained in HMZ *aip^wh86^* embryos from HET parents until around 5 dpf [41], aligning with the emergence of the phenotypes in mutant larvae. RNA adaptation via compensatory mechanisms [58] was considered but deemed incomplete or absent, as the *aip^wh86^* larvae had clear morphological deterioration, also shown in this study and complementary studies [42].Another study found similar patterns in the original *aip* mutant lines (on a TL genetic background), with morphological phenotypes indistinguishable between *aip^wh86^* and WT larvae up until 5 dpf [41]. They also found that these phenotypes resulted in early lethality in the HMZ *aip^wh86^* larvae, but that such early lethality was not seen in previous studies of HMZ *ahr2* mutant larvae (*ahr2^hu3335^*), suggesting a role for other developmental processes interacting with the *aip*. For all phenotypes observed in the mutant, the quick emergence and increased penetrance from 5–9 dpf lend credence to the multi-organ and tissue-level effects that the *aip* has during development.

The connection between thalassemias and sickle cell disease with altered globin gene expression is well established in vertebrates [59,60]. Often, this is due to changes in the abundance of either alpha or beta globins in relation to each other, the overall abundance of the various globins, or the repression and activation of specific globins at specific times. While mutants show an appropriate homeostatic ratio of *hbae1* and *3*, and *hbbe1* and *2*, which is vital to proper development [22,23], by 7 dpf the mutants display a disrupted balance and prolonged overexpression of *hbae4* and *hbbe3*, potentially leading to too much free heme and increasing the potential development of hematopoietic pathologies [23,61]. Similarly, the premature expression of *hbae5* in mutant larvae, which shouldn’t be expressed until ~18 dpf [22,23], demonstrates late-stage embryonic/early larval hematopoietic dysregulation. This combination of early and later-stage embryonic hematopoietic dysregulation suggests that the *aip* has non-specific and broad impacts on heme synthesis and hemoglobin subunit formation. To our knowledge, this study represents the first known linkage of dysregulated globin synthesis to an *aip* mutation.

Alterations in transcripts involved in hematopoietic signaling and differentiation of early erythrocyte progenitors are known to lead to various developmental consequences and pathologies, including malignancies of the blood and anemias [62,63,64]. Improper expression of the hematopoietic transcription factors *gata1* and *klf1*, which were both overexpressed at 5 and 6 dpf in mutant larvae, can lead to catastrophic blood and vascular pathologies, especially in embryonic development [24,65]. The overabundance of *alas2*, which encodes an enzyme responsible for catalyzing the first and rate-limiting step in the heme biosynthetic pathway, at day 6 but not day 7, suggests a potential feedback mechanism, attempting to control the overactivation of the heme synthesis pathway. Consequentially, if this transcript is overabundant, an overproduction of heme or intermediate metabolites can occur [39], resulting in cytotoxicity, reactive oxygen species (ROS) generation, lipid peroxidation, and membrane disruption [61,66]. The gene *epoa*, if overexpressed, as seen in the mutant larvae at both time points, can induce hemangioblasts and hematopoietic stem cells (HSCs) to favorably differentiate into mature erythrocytes rather than maintaining stem cell populations, potentially leading to polycythemia [31,67]. Though *mmp9*, a known marker for myeloid differentiation, had transcriptional abundance in the mutant comparable to that of WT, more gene markers are needed to confidently conclude that myeloid differentiation and early immune system regulation are preserved in the mutant.

The increased hemoglobin content observed in the cardiac and liver region after O-dianisidine staining suggests either increased red blood cell formation or increased hemoglobin content per red blood cell, both of which are potentially pathological [35]. Polycythemia, defined as the overabundance of red blood cells, can cause increased clotting or burden on the cardiovascular system, causing multi-organ damage and developmental decline [68,69]. The pooling of the stain in the cardiac and liver region could be due to many pathological outcomes of too many blood cells, such as decreased venous drainage or trapping of the overabundant erythrocytes in the hepatic sinusoidal capillaries [70]. Alternatively, increased hemoglobin content can cause increased erythrocyte mass and viscosity, increasing the chances of blood clotting and hemorrhages [71,72]. If erythrocytes are dying, increased levels of cell-free hemoglobin can lead to oxidative stress and tissue damage [73].

Hematopoietic stem cell dysregulation was observed in the genes *tal1*, *c*, and *runx1*, which are responsible for HSC differentiation, maintenance, and survival [28,74]. The overabundance of these transcripts suggests that *aip^wh86^* larvae not only show dysregulation of downstream erythroid populations but also dysregulation of early hematopoietic pathways involved in HSC populations and core hematopoietic pathways. The increased abundance of these transcripts could be due to direct Aip- or Hif-mediated control, but also could be a compensatory mechanism to keep up with the constant overproduction of heme and erythrocytes [75,76]. Connecting these overabundant transcripts to an *aip* mutation in a developing model provides insight into how the *aip* may affect both various upstream and downstream steps of developmental processes, like hematopoiesis.

While day 6 represents DEGs with specific linkage to perturbed developmental processes, day 7 shows broader changes in metabolic processes concordant with systemic physiological collapse. Many of these pathways induce oxidative stress when dysregulated, potentially causing cytotoxicity and developmental abnormalities. For example, when perturbed, aromatic amino acid metabolism has been linked to hepatic damage and irregular liver development [77]. Other such metabolic effects have been observed in zebrafish embryos exposed to other xenobiotics, such as lead and halogenated bisphenols, leading to edema, swim bladder malformities, and bent axes [78,79]. With these broad metabolic systems perturbed, vital anabolic or catabolic processes needed for development are altered, affecting the way the embryo develops. The molecular role of *aip* in these processes is unknown, but various possibilities exist. First, *aip* could interact with and stabilize these pathways at this particular time point. When the transcript is reduced, these pathways falter quickly. Second, the lack of *aip* transcript in zebrafish in earlier pathways at day 6 could cascade into systemic failure of broader physiological and metabolic systems. For example, the changes in hematopoiesis seen in *aip^wh86^* larvae at day 6 could lead to increased oxidative stress and metabolic dysfunction, as shown in day 7. Improper blood flow, either due to clotting or anemia, could cause systemic tissue damage. If future studies can fully rescue the early hematopoietic phenotype, the subsequent effect on downstream metabolic transcriptional changes and phenotypes could highlight whether the Aip has a primary role, through direct binding with proteins or regulators of these pathways, or a cascading secondary role due to earlier pathway perturbation.

The transcriptional profiles and morphological phenotypes of *aip^wh86^* larvae demonstrate a perturbation in early hematopoietic pathways, putatively linked via the Hifα pathway. A previous study in zebrafish reported perturbation of similar hematopoietic pathways after activation of the *ahr* [80], but we believe this is the first report of an *aip* mutant displaying this phenotype. Other bHLH-PAS proteins outside of Hifα have also been linked to hematopoietic cell fate and development. In one study, zebrafish knockouts of the class I bHLH-PAS protein npas4l (cloche) displayed decreased hematopoietic transcriptional markers [81]. Similar findings were seen in double knockout zebrafish mutants in the class II bHLH-PAS proteins, Arnt 1 and 2 [28]. Due to the shared hematopoietic phenotypes in Aip knockouts and Aip’s known binding to the Ahr, which is a bHLH-PAS protein, the possibility that the Aip acts as a chaperone for other bHLH-PAS proteins must be recognized. The *aip* also has a known role in maintaining proteasome activity and assisting in the ubiquitination of proteins [82]. If proteins that require constant degradation to maintain homeostatic balance, like Hifα proteins, are not degraded due to dysregulated proteostasis, this could lead to an increased nuclear abundance and activation. This proposed perturbation of the Hifα cycle is further supported by the overexpression of *egln2*, which is responsible for the initial oxygenation step in the ubiquitination process of Hifα [29,30,67,83,84]. The overexpression of the *egln2* transcript suggests a feedback response to elevated or overactivated Hifα levels. If Hifα escapes degradation and successfully translocates to the nucleus, it heterodimerizes with the same binding partner as the Ahr, Hifβ/Arnt. Even if excess Hifα makes it into the nucleus, it is in constant competition with the Ahr to bind to free Arnt, creating another source of control to prevent Hifα overactivation. It has been established that a lack of functional Aip results in both increased degradation and nuclear localization of the Ahr, as well as altered Arnt activity and abundance [6,19,67,85]. Hifα proteins regulate many hematopoietic transcription factors during their role in regulating the maintenance of HSCs and the differentiation of erythrocytes, usually through sensing of hypoxia.

Previous studies of zebrafish with a null mutation in the *vhl* gene, which is involved in the degradation of Hif proteins after oxygenation by *egln2*, reported morphological and transcriptional phenotypes similar in type and timing to those of our *aip^wh86^* fish. Upon *vhl* knockout, mutant larvae presented with a distorted liver, deflated swim bladder, early mortality from 8–11 dpf, and overexpression of Hif-regulated genes [55,74,86]. In one such study, pharmacological inhibition of *hif2α* RNA yielded a slight rescue of observed phenotypes. Morphologically, this decreased the incidence of phenotypes observed and delayed early mortality, while also showing decreases in overabundant transcripts compared to the unexposed *vhl* mutants. This similarity in morphological and transcriptional phenotypes suggests that these two different mutants may share an overactivation of the Hifα pathway. Similar rescue studies were performed on *aip^wh86^* larvae with the same *hif2α* inhibitor, compound 76, at similar concentrations of 10 and 100 μM, but, in contrast to the results with *vhl* mutants, the rescue of morphological endpoints was not observed in our study. Small transcriptional changes were observed in the treated *aip^wh86^* larvae compared to the untreated. Decreased expression of *gata1*, *klf1*, and *epoa*, and increased expression of *egln2* were observed. The globin and heme-related transcripts remained unchanged. This demonstrated that the compound was able to slightly rescue certain transcripts through apparent Hif inhibition, but they were still above standard WT levels. The lack of morphological rescue and the increased abundance of *egln2* transcripts was unexpected, but could occur through feedback loop activation after Hif inhibition, or Hif-1α compensation [87]. In vivo rescue experiments in mutants are notoriously difficult, especially with a gene responsible for such diverse phenotypes. Western blotting to validate Hif2α inhibition, along with dual inhibition of Hif1α, is needed to determine if Hif2α is the causative pathway for the phenotypes observed, versus Hifα isoforms sharing a regulatory role in this phenotype.

Although the canonical function of the Aip is known to be as a chaperone and stabilizer of the Ahr complex, the relationship between the two proteins, which could affect the endogenous roles and xenobiotic response of the Ahr, remains poorly understood. A better understanding of the developmental role of the Aip provides more insight into how the Ahr complex functions as a whole, with respect to both its endogenous roles in both development and xenobiotic response. Adjacent studies have shown that *aip^wh86^* larval zebrafish display decreased sensitivity to dioxin-like chemicals (TCDD and PCB126), and both decreased and increased sensitivity to various PAHs [41,42]. This illustrates the complex relationship between the Aip and the Ahr that affects its downstream xenobiotic response and transcriptional control; however, whether the stability of the complex, differential conformational shape changes, or both, are responsible, is unknown. Altered xenobiotic response also suggests that Ahr-mediated endogenous pathways could be disrupted, potentially causing inhibition or overactivation, and leading to toxicity and early lethality. The Ahr has been linked to hematopoiesis, with Ahr ligands known to be Ahr inducers, like TCDD, showing epidemiological linkage to leukemias [88]. Even non-Ahr-binding ligands that are hematotoxic, like benzene, have shown decreased toxicity in Ahr null mice [89]. Other studies have linked Ahr antagonism to over-proliferation of HSC populations in mice and human cells, with the hypothesis that the Ahr acts as a negative regulator of proliferation and helps maintain quiescence of HSCs during development [90,91]. However, observing that *aip* mutants are directly linked to these phenotypes in zebrafish, while *ahr* mutant zebrafish lines do not, demonstrates how other proteins within the *ahr* complex can affect hematopoiesis and embryonic development. Additionally, Ahr dysregulation could feed into Hif overactivation, as both pathways bind to the same heterodimerization partner. Further investigation into the concordance between Ahr activation/inhibition and *aip* knockout phenotypes is needed to better understand the interplay between these proteins and the resulting toxicological or developmental outcomes.

## 5. Conclusions

Our results suggest that loss of Aip disrupts Hifα signaling pathways, possibly through crosstalk with Ahr. However, the phenotypes observed in the *aip^wh86^* larvae may be distinct from those of both Ahr and Hifα. The Aip protein has been shown to bind to other receptors and protein complexes, and given the observed diversity in morphological and transcriptional changes, it may affect multiple pathways simultaneously. The significant decrease in Hifα and hematopoietic transcripts after Hif2α pharmacological inhibition, but no rescue in morphology, supports the notion of Hif2α involvement in the developmental decline of *aip^wh86^* larvae. However, many of these transcriptional targets are not exclusively regulated by Hif2α, warranting future studies investigating other proteins, such as Hif1α or Vhl. Other pathways associated with the non-canonical activation of Hifα, such as the jak/stat3 and glucocorticoid signaling pathways, could be involved in the overactivated hematopoiesis phenotype observed in the *aip* mutants [92,93]. Pharmacological inhibition or morpholino knockouts could provide deeper resolution into the impact of these pathways on aip mutant phenotypes.

The strength of this study lies in understanding the molecular changes occurring in vivo, with morphologically anchored transcriptomics. Different RNA sequencing techniques, such as scRNA seq or spatial RNA seq, could provide information regarding specific cell/tissue types affected by an *aip* mutation, with particular focus on hematopoietic and hepatic lineages. By providing a baseline characterization of the consequences of *aip* loss-of-function in zebrafish larvae, we not only aim to determine its role in xenobiotic response and the Ahr, but also hope to better understand a small piece of the most complex time in an organism’s life—development.

## Data Availability

The original data presented in the study are openly available in NCBI Gene Expression Omnibus Accession at [GSE344189].

## Supplementary Materials

The following supporting information can be downloaded at https://www.mdpi.com/article/10.3390/toxics14090807/s1, Figure S1: H&E Stain; Figure S2: Liver size timecourse comparison; Figure S3: RNASeq PCA; Figure S4: DMOG O-Dianisidine; Figure S5: Hif Target Primer Validation; Table S1: Taqman Genotyping Probes; Table S2: RT-qPCR Primers; Table S3: Hematopoietic Gene Expression. RNA sequencing DEG Excel Files are available in Supplementary Data.

## Abbreviations

The following abbreviations are used in this manuscript:

Ahr	Aryl Hydrocarbon Receptor
AGM	Aorta Gonad Mesonephros
AIP	Ahr Interacting Protein
ALM	Anterior Lateral Mesoderm
CHT	Caudal Hematopoietic Tissue
CYPs	Cytochrome P450
DEG	Differentially Expressed Gene
dpf	Days Post Fertilization
EM	Embryo Media
ENSDART	Ensembl Transcript ID
FIPA	Familial Isolated Pituitary Adenoma
PFA	Paraformaldehyde
gDNA	Guide DNA
HET	Heterozygous
HMZ	Homozygous
HSC	Hematopoietic Stem Cell
ICM	Intermediate Cell Mass
NBF	Neutral Buffered Formalin
PCA	Principal Component Analysis
QC	Quality Control
ROS	Reactive Oxygen Species
RT	Room Temperature
SARL	Sinnhuber Aquatic Research Laboratory
sgRNA	Single Guide RNA
TPR	Tetratricopeptide Repeat
UP	Ultra-Purified Water
VDL	Vet Diagnostic Laboratory
WT	Wild Type
XREs	Xenobiotic Response Elements
ZEG	Zebrafish Embryonic Genotyper
