# Supplementary material for: aip Mutant Zebrafish Display Morphological and Transcriptional Changes Associated with Stress-Induced Hematopoiesis
Source: Toxics. 2026 Sep 11;14(9):807. doi: 10.3390/toxics14090807 (PMC13611658; doi:10.3390/toxics14090807)
Supplement: Supplementary file 1 [file toxics-14-00807-s001.zip › Supplementary Figures and Tables/Figure S1.pdf]

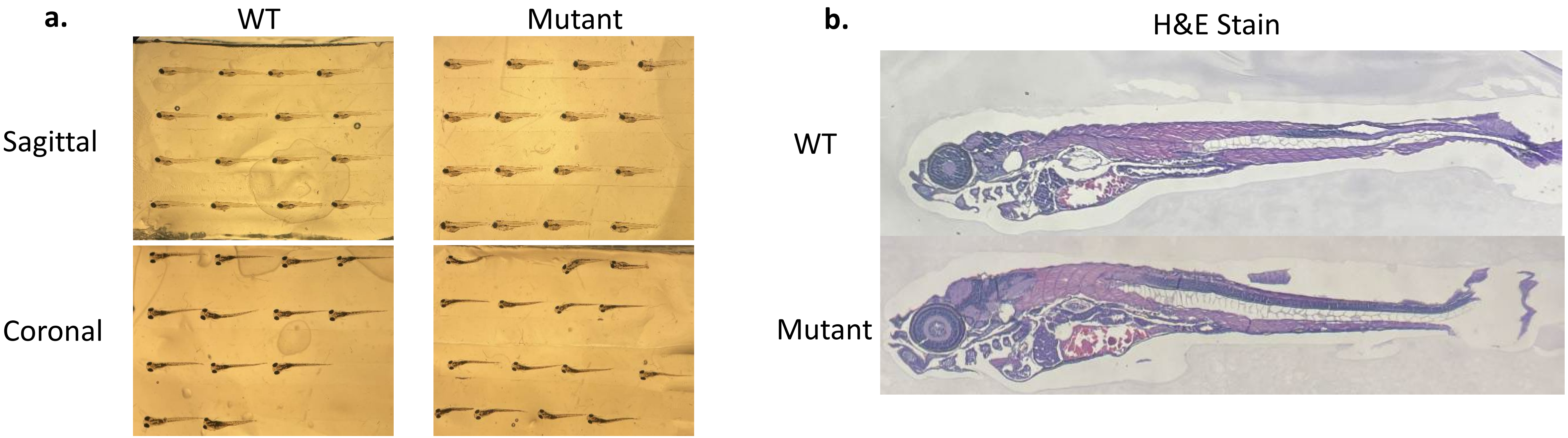
a.
WT
Mutant
Sagittal
Coronal
b.
H&E Stain
WT
Mutant
