# Supplementary material for: aip Mutant Zebrafish Display Morphological and Transcriptional Changes Associated with Stress-Induced Hematopoiesis
Source: Toxics. 2026 Sep 11;14(9):807. doi: 10.3390/toxics14090807 (PMC13611658; doi:10.3390/toxics14090807)
Supplement: Supplementary file 1 [file toxics-14-00807-s001.zip › Supplementary Figures and Tables/Figure S2.pdf]

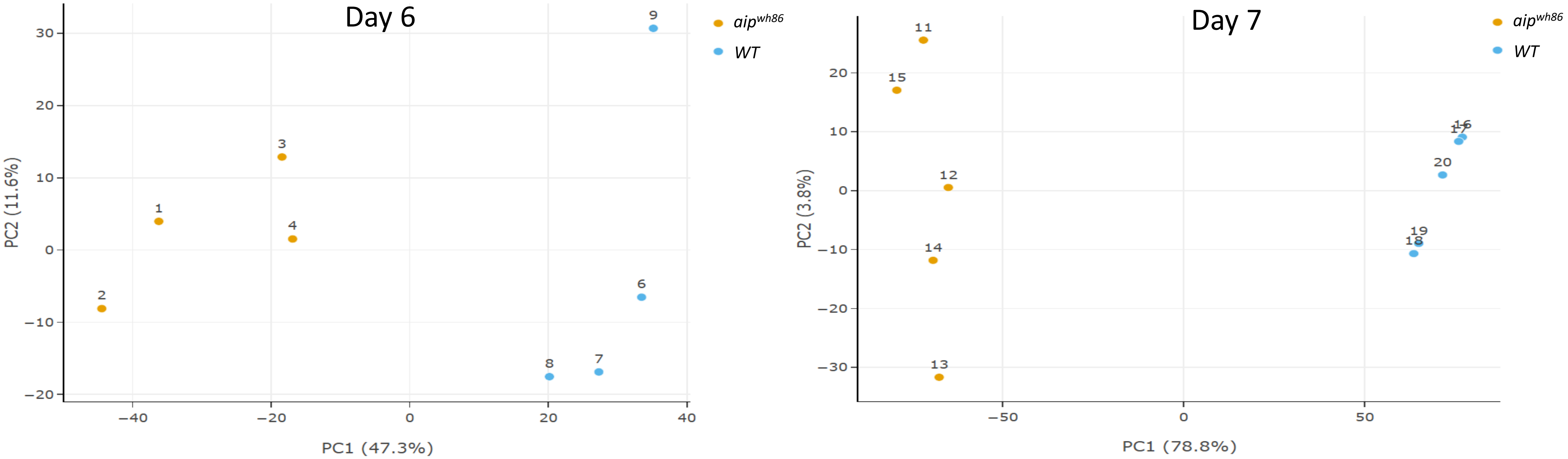

Day 6
aip^wh86
WT
PC2 (11.6%)
PC1 (47.3%)
30
20
10
0
−10
−20
−40
−20
0
20
40
1
2
3
4
6
7
8
9
Day 7
aip^wh86
WT
PC2 (3.8%)
PC1 (78.8%)
20
10
0
−10
−20
−30
−50
0
50
11
12
13
14
15
16
17
18
19
20
