# Supplementary material for: aip Mutant Zebrafish Display Morphological and Transcriptional Changes Associated with Stress-Induced Hematopoiesis
Source: Toxics. 2026 Sep 11;14(9):807. doi: 10.3390/toxics14090807 (PMC13611658; doi:10.3390/toxics14090807)
Supplement: Supplementary file 1 [file toxics-14-00807-s001.zip › Supplementary Figures and Tables/Figure S5.pdf]

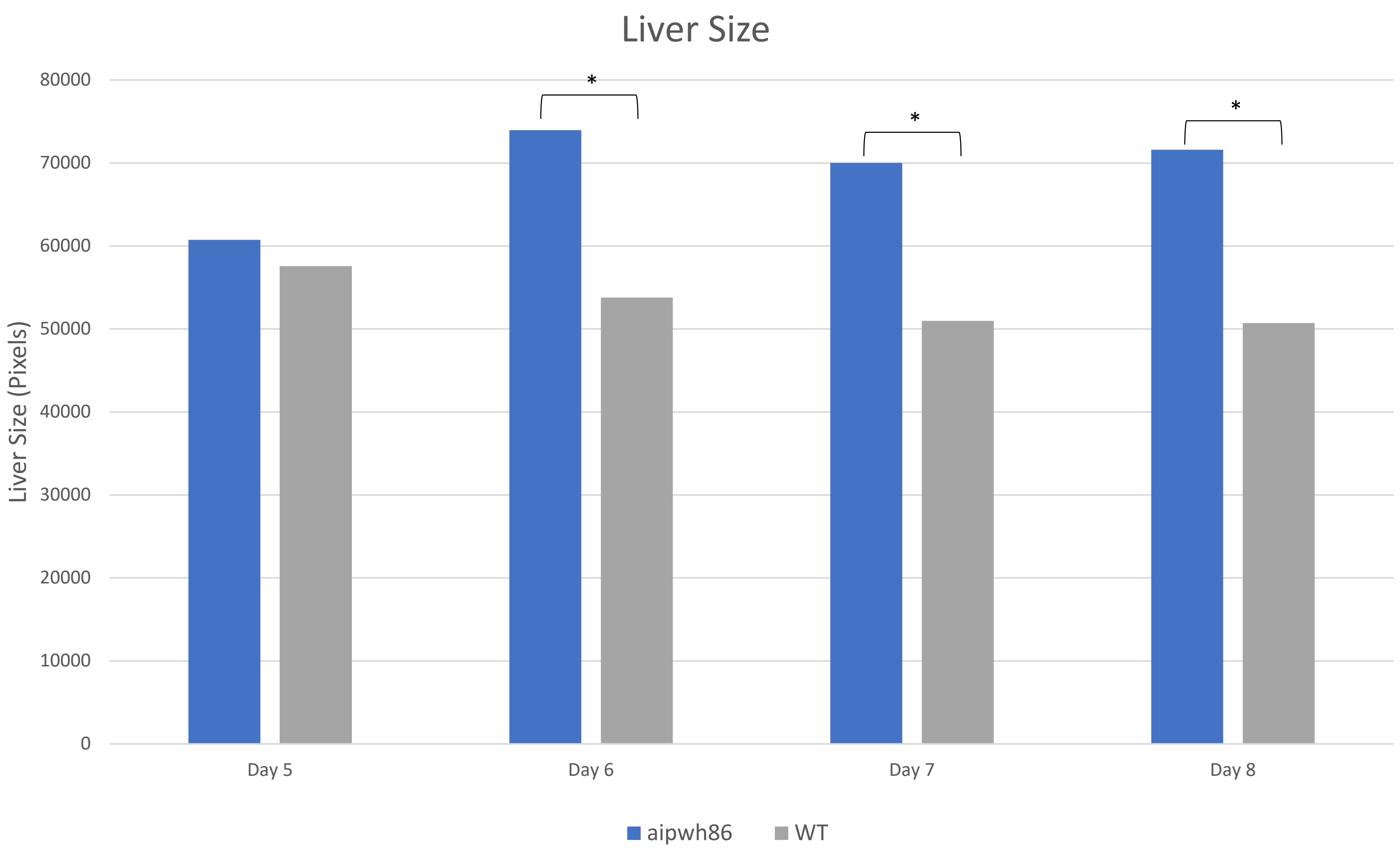

Liver Size
Liver Size (Pixels)
80000
70000
60000
50000
40000
30000
20000
10000
0
*
*
*
Day 5
Day 6
Day 7
Day 8
aipwh86
WT
