# Supplementary material for: aip Mutant Zebrafish Display Morphological and Transcriptional Changes Associated with Stress-Induced Hematopoiesis
Source: Toxics. 2026 Sep 11;14(9):807. doi: 10.3390/toxics14090807 (PMC13611658; doi:10.3390/toxics14090807)
Supplement: Supplementary file 1 [file toxics-14-00807-s001.zip › Supplementary Figures and Tables/Table S1.pdf]

aip^wh86^ Genotyping	
TaqMan Fwd	5’-TGTGTGGGAGCAAGTGGTTAC-3’
TaqMan Rev	5’-ACAGCTAGAAATGTCACTGACCTTAAC-3’
TaqMan WT Probe	5’-TTCCCCCTCTTTCATAGTG-3’
TaqMan Mutant Probe	5’-ATTTCCCCCTTTCATAGTG-3’
