# Supplementary material for: aip Mutant Zebrafish Display Morphological and Transcriptional Changes Associated with Stress-Induced Hematopoiesis
Source: Toxics. 2026 Sep 11;14(9):807. doi: 10.3390/toxics14090807 (PMC13611658; doi:10.3390/toxics14090807)
Supplement: Supplementary file 1 [file toxics-14-00807-s001.zip › Supplementary Figures and Tables/Table S2.pdf]

Gene	Forward Primer Sequence	Reverse Primer Sequence	Product Length
**hbae1**	F: 5’ CTTTGTTCCCCGCCGATTTC 3’	R: 5’ CAGAGAGTTTGGAGCACAGT 3’	124 bp
**hbbe1**	F: 5’ TGGCAAGGTGTCTCATCGTG 3’	R: 5’ GTTTTACCGTGGGCAGCAAC 3’	115 bp
**Mmp9**	F: 5’ TGCCAACGAAACCTTCTCCA 3’	R: 5’ CCTTGATTTGGCAGGCATCG 3’	90 bp
**egln2**	F: 5’ TCTGGTCTGATCGGCGAAAC 3’	R: 5’ TTCTCCTTCGCTTCAGCTCG 3’	109 bp
**klf1**	F: 5’ TCATCTCAAAGCACACCTCCG 3’	R: 5’ GTCTCTTCATGTGTAACGCCAG 3’	200 bp
**alas2**	F: 5’ GGACTGCCTCTGAATGGACC 3’	R: 5’ AGACTTCTCCCACTCGCTCA 3’	90 bp
**epoa**	F: 5’ GCATCAGACAAGTGCTGCG 3’	R: 5’ AGACAGGTGCATTGGCGAG 3’	168 bp
**gata1**	F: 5’ TAGACACAGTCCAGTTCGCC 3’	R: 5’ GTGGATGTGGGGTTGTAGGG 3’	114 bp
**tal1**	F: 5’ TGGCTCCATGCACACACTAA 3’	R: 5’ CAGCGGAAATTGCTCGGATTT 3’	158 bp
**runx1**	F: 5’ GAAGTACTGTCCGACCACCC 3’	R: 5’ CCAAGGGCAACCACCTTGA 3’	128 bp
**cmyb**	F: 5’ AGAAGTGCCCCCACTTTTCTT 3’	R: 5’ CTGAGGTCGAAGAGCAAGGT 3’	186 bp
**β-actin**	F: 5’ GATCAAGATCATTGCCCCACC 3’	R: 5’ GAGTCGGCGTGAAGTGGTAA 3’	179 bp
