# Supplementary material for: aip Mutant Zebrafish Display Morphological and Transcriptional Changes Associated with Stress-Induced Hematopoiesis
Source: Toxics. 2026 Sep 11;14(9):807. doi: 10.3390/toxics14090807 (PMC13611658; doi:10.3390/toxics14090807)
Supplement: Supplementary file 1 [file toxics-14-00807-s001.zip › Supplementary Figures and Tables/Table S3.pdf]

Day 6 vs Day 7 Log_2FC RNA Seq

Genes	Day 6	Day 7
mmp9	2.90	5.27
epoa	1.76	2.59
hbae1	2.20	1.89
hbbe1	2.14	1.88
alas2	2.37	2.04
egln2	1.20	1.27
klf1	1.98	1.35
